# The impact of “*To Err Is Human*” on patient safety in anesthesiology. A bibliometric analysis of 20 years of research

**DOI:** 10.3389/fmed.2022.980684

**Published:** 2022-11-16

**Authors:** Christopher Neuhaus, Petra Grawe, Johan Bergström, Michael St.Pierre

**Affiliations:** ^1^Department of Anesthesiology, Heidelberg University Hospital, Heidelberg, Germany; ^2^Department of Anesthesiology, University Hospital Erlangen, Erlangen, Germany; ^3^Division of Risk Management and Societal Safety, Lund University, Lund, Sweden

**Keywords:** patient safety, safety, human factors, anesthesiology, safety research, bibliometrics, bibliometric analysis

## Abstract

**Background:**

Patient safety gained public notoriety following the 1999 report of the Institute of Medicine: *To Err is Human – Building a Safer Health System* which summarized a culminated decades' worth of research that had so far been largely ignored. The aim of this study was to analyze the report's impact on patient safety research in anesthesiology.

**Methods:**

A bibliometric analysis was performed on all anesthesiologic publications from 2000 to 2019 that referenced *To Err Is Human*. In bibliometric literature, references are understood to represent an author's conscious decision to express a relationship between his own manuscript and the cited document.

**Results:**

The anesthesiologic data base contained 1.036 publications. The journal with the most references to the IOM report is Anesthesia & Analgesia. By analyzing author keywords and patterns of collaboration, changes in the patient safety debate and its core themes in anesthesiology over time could be visualized. The generic notion of “error,” while initially a central topic in the scientific discourse, was subsequently replaced by terms representing a more granular, team-oriented, and educational approach. Patient safety research in anesthesia, while profiting from a certain intellectual and conceptual head start, showed a discursive shift toward more managerial, quality-management related topics as observed in the health care system as a whole.

**Conclusions:**

Over the last 20 years, the research context expanded from the initial focus set forth by the IOM report, which ultimately led to an underrepresentation of research on critical incident reporting and systemic approaches to safety. Important collaborations with safety researchers from outside of health care dating back to the 1990's were gradually reduced, while previous research within anesthesiology was aligned with a broader, more managerial patient safety agenda.

## Introduction

Anesthesiology is generally acknowledged to have achieved order of magnitude improvements in safety in the comparatively short time span of a few decades. While much of that success is commonly attributed to technological innovation, a more complex story fortifies its role as vanguard of the modern patient safety movement (1) beginning with the seminal 1978 study by Cooper et al. (2).

In the wake of the 1979 nuclear disaster of the Three Mile Island reactor in Pennsylvania (and various other highly visible accidents) the multidisciplinary research tradition of safety science gained momentum, generating a plethora of concepts about work in complex sociotechnical systems and a new understanding of “human error” (3). While generally unrecognized, anesthesiology was likely the first healthcare specialty to tap into this rich body of knowledge generated through research methodology foreign to medicine. Ultimately, this resulted in practice innovation and substantial progress in anesthetic patient safety that by far exceeded the benefits of technology alone (4).

On a broader scale, patient safety gained public notoriety following the 1999 report of the Institute of Medicine (IOM): *To Err is Human – Building a Safer Health System* (5) which summarized a culminated decades' worth of research that had so far been largely ignored. The comprehensively researched information, in combination with alarming “body counts” (6) and interpretations by the IOM shocked the public, elevated the occurrence of patient harm to the level of an epidemic health crisis, spawned research on patient safety, and initiated a discursive change in the patient safety debate.

On the occasion of the IOM report's 20th anniversary, we previously analyzed the academic impact the report had on global efforts in patient safety research (7). As anesthesiology initially sought the cooperation with human factor specialists, it is conceivable that the report created an academic momentum in this particular specialty different to the rest of the medical field. The aim of this study was to gain a comprehensive and systematic understanding of the academic impact and momentum the IOM report created within anesthesiology by applying bibliometric methods. This assumes that any publication referencing *To Err Is Human* is most likely conceptually or discursively influenced by the IOM report.

## Materials and methods

### Bibliographical methods

Methodologically, it is possible to assess and measure scientific impact by applying bibliometrics to scientific publications stored or indexed in big bibliographic databases (8). Bibliometrics provides robust analyses of large amounts of published research by applying mathematical and statistical methods in the study of the use of documents and publication patterns. The two main methods commonly applied in bibliometric studies are performance analysis and science mapping (8, 9): Performance analysis (10) aims to evaluate the research and publication performance of scientific actors (i.e., individuals, institutions, countries) by analyzing bibliographic coupling (11) and co-citation patterns (12).

Science mapping uses bibliometric methods to assess the social, intellectual, and conceptual structure of a research field and describe its knowledge base. This is done by analyzing the publications' meta data about authors, institutions, and countries (13, 14), the co-citation networks among publications (12) and by means of co-words analysis (15). We described the bibliometric methods applied to this study in great detail in a previous publication (7). Based on the study design, the study was exempted from approval by the University of Heidelberg Ethics Review Board.

### Data collection and analysis

The data for this study were retrieved from Scopus (www.scopus.com, Elsevier B.V., Amsterdam, NL) on July 7th, 2020. By using the “references” filter and the query “to err is human” in the Scopus database, all documents bibliographically coupled to the IOM report from 1999–2019 were retrieved. The search results returned all essential bibliographic information (e.g., title, author's names and affiliations, abstracts, keywords, references) and were exported and stored in two different formats: BibTeX –files (^*^.bib) for import into the bibliometric application Biblioshiny (Bibliometric analysis program “Bibliometrix,” designed by Aria M. and Cuccurullo C. 2017) and ^*^.ris-files for import into the citation management software Endnote X8 (Clarivate Analytics). Publications from the field of anesthesiology were identified in Endnote X8 using the query „anesthe^*^” and „anaesthe^*^”. As Biblioshiny cannot preprocess imported data, the publications from the field of anesthesiology identified in Endnote served as basis for further manual selection within the BibTeX–files. For this purpose, the ^*^.bib-files were imported into the unicode editor Texmaker (https://www.xm1math.net/texmaker/), which allowed to manually delete files not belonging to the database.

Analyses were carried out using the open-source R-based tool bibliometrix (16) and its web user interface biblioshiny (17). Due to a large variety in reference notation (e.g., the IOM report Crossing the Quality Chasm (2001) is referenced with 2.322 (!) different entries), biblioshiny's query results for the most locally cited references had to be completed manually. For that purpose, the data base was searched for the exact title of biblioshiny's suggested top results and additional references were identified. References pointing toward the most relevant safety scientists were found by searching the data base for their family names and by manually identifying documents with the correct surname.

**Figure 1:**
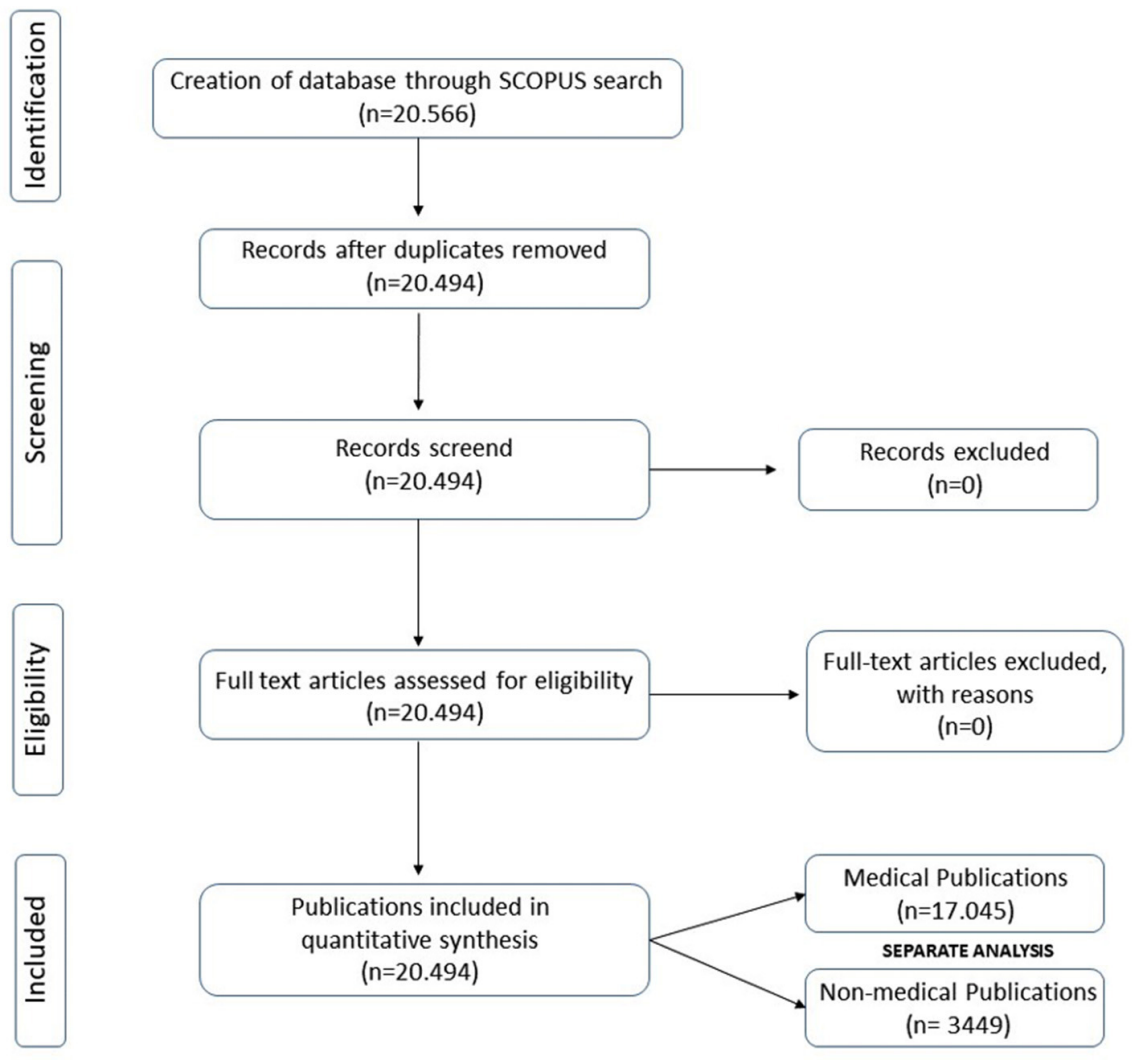
SRQR flow diagram of the identification, screening and inclusion of source documents. Publications were subsequently separated in to medical and non-medical documents for further analysis.

The workflow adheres to the applicable EQUATOR guidelines (Standards for Reporting Qualitative Research SRQR) and is illustrated in Figure 1.

## Results

**Figure 2:**
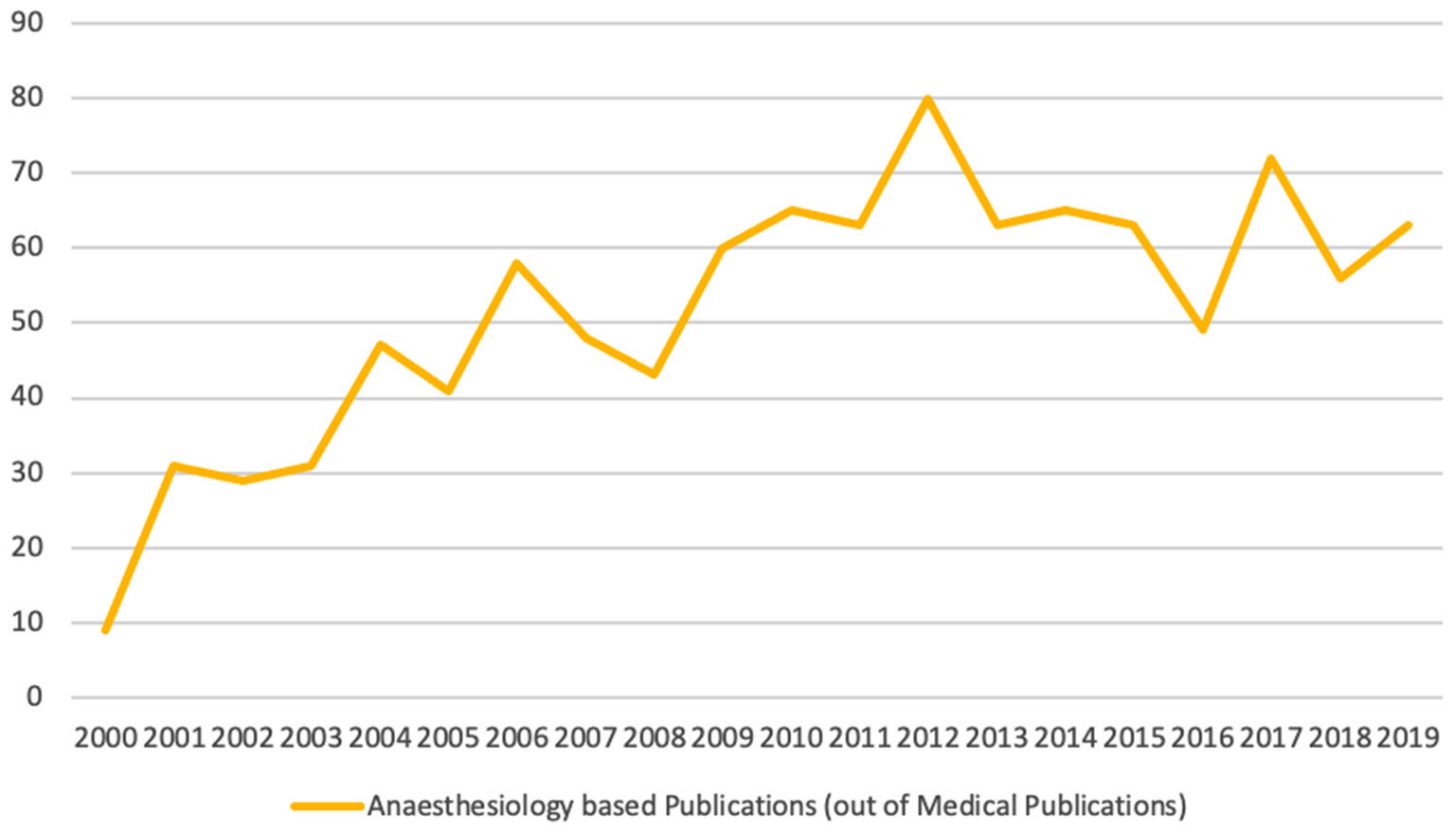
Number of anesthetic publications per year citing *To Err Is Human*.

The Scopus database search resulted in 20.566 matching documents. The anesthesiologic data base contained 1.036 publications (Table 1). Our analysis covers the scientific production period of 2000–2019. The number of publications citing the IOM report increased steadily from 2000 to 2012. After 2012, we observed a declining trend (Figure 2).

**Table 1:** Main bibliographical information.

**Description**	**Explanation**	**Results**
**Main information**		
Sources (Journals, Books, etc)		387
Documents	Total number of documents	1,036
Documents sub-period 1	Documents from 2000 to 2004	147
Documents sub-period 2	Documents from 2005 to 2009	250
Documents sub-period 3	Documents from 2010 to 2014	336
Documents sub-period 4	Documents from 2015 to 2019	303
Average citations per documents	Average number of citations in each article	24,68
Average citations per year per doc		1,926
References	Total number of references in all documents	38,713
**Document types**		
Article		550
Book		3
Book chapter		41
Conference paper		34
Editorial		78
Letter		26
Note		16
Review		279
Short survey		9
**Document contents**		
Keywords plus (ID)	Total number of phrases that frequently appear in the title of an article's references	4,692
Author's keywords (DE)	Total number of keywords (MeSH etc.)	1,468
**Authors**		
Authors	Total number of authors	3,242
Author Appearances	The authors' frequency distribution	3,878
Authors of single-authored documents	The number of single authors per article	178
Authors of multi-authored documents	The number of authors of multi-authored articles	3,064
**Authors collaboration**		
Single-authored documents		201
Documents per Author		0.32
Authors per Document		3.13
Co-Authors per Documents		3.74
Collaboration Index	Number of authors that contribute to a multi-authored article	3.67

### Highly influential authors and papers

Table 2 lists the twenty most influential authors, sorted both in terms of total number of publications they authored or co-authored and in terms of number of citations authors received within our database (i.e., locally cited authors). Only three authors published more than 10 articles, while 87% (*n* = 2.877) published only one article. The author with the biggest academic impact (22 articles, h-index 12, g-index 21) is Merry AF.

**Table 2:** The 20 most relevant authors with the number of articles citing *To Err is Human* and the 20 most locally cited authors from 2000 to 2019.

	**Most relevant authors**	**Articles**	**h-index**	**g-index**	**Most local cited authors**	**Number of citations**
1	Merry AF	22	12	21	Gaba DM	613
2	Webster CS	20	12	20	Leape LL	430
3	Pronovost PJ	13	10	13	Cooper JB	354
4	Rall M	9	5	9	Bates DW	323
5	Sevdalis N	8	7	8	Merry AF	314
6	Staender S	8	4	8	Brennan TA	291
7	Barach P	7	5	7	Howard SK	278
8	Brattebø G	7	5	7	Flin R	265
9	Manser T	7	4	7	Webster CS	254
10	Martinez EA	7	5	7	Runciman WB	240
11	Weinger MB	7	6	7	Helmreich RL	237
12	Cooper JB	6	4	6	Reason J	229
13	Espin S	6	6	6	Vincent C	220
14	Gaba Dm	6	5	6	Pronovost PJ	207
15	Lagasse RS	6	3	6	Thomas EJ	206
16	Lingard L	6	6	6	Salas E	204
17	Mahajan RP	6	4	6	Cullen DJ	186
18	Clergue F	5	3	5	Sexton JB	185
19	Dutton RP	5	3	5	Berry WR	183
20	Fleisher LA	5	4	5	Gawande AA	174

The three editors of *To Err is Human*, Kohn LT, Corrigan JM, and Donaldson MS were excluded from the list of locally cited authors.

**Figure 3:**
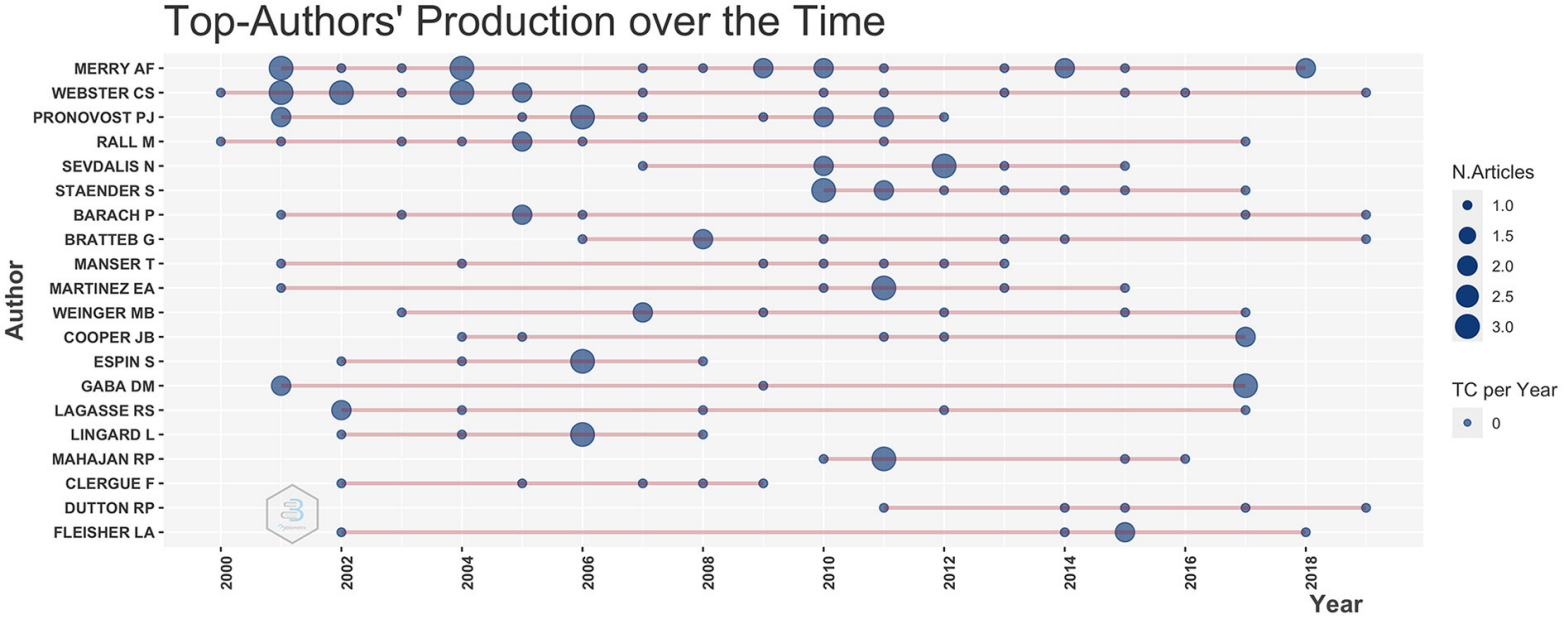
The 20 top-author's production over time. The line represents an author's timeline. The bubble size is proportional to the number of documents published, and the color intensity is proportional to the total citations per year the documents collected.

Six of the 20 most influential authors are among the most locally cited authors (within the dataset). Some authors, while often cited, published less documents referencing *To Err is Human* (e.g., Gaba DM, who is #1 of locally cited authors but only #14 of the most relevant authors in the database). Other authors are cited most likely for their theoretical framework that help understand patient safety (e.g., Vincent C or Reason J). Both authors, Merry AF und Webster CS, showed near constant annual publications, while Pronovost PJ was active in anesthesiology from 2000 to 2012, with a highly cited article in 2006 (Figure 3).

### Publications citing the IOM report

**Figure 4:**
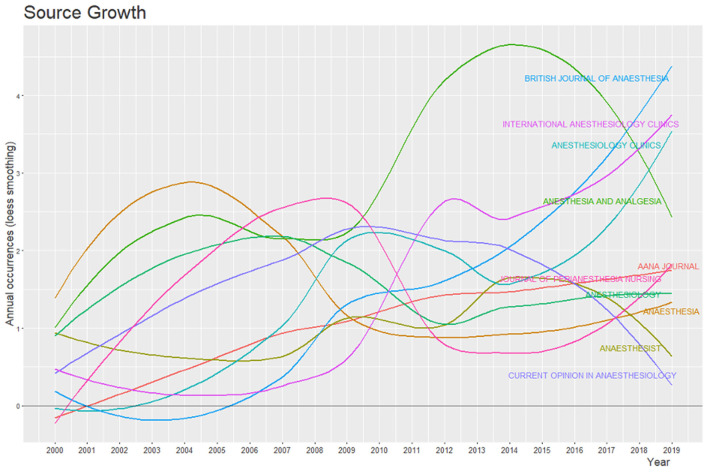
Annual occurrences of publications in the most relevant sources. To enhance legibility of the plot, the number of source dynamics was restricted to the 10 most relevant journals.

The 1.036 documents included in the bibliographic collection were published in 387 sources (Table 2). The majority of these were published as peer reviewed articles (53%), followed by reviews (27%), books and book chapters (4%). The remaining documents included conference papers, short surveys and editorials. The most references to the IOM report were found in the journal Anesthesia & Analgesia. The most impactful journal was Anesthesiology with a total of 1.893 citations received (Table 3). The dynamics of publications over time for journals is shown in Figure 4. It visualizes the growing relevance of Anesthesia & Analgesia between 2009 and 2015. Starting in 2017, three other journals surpassed Anesthesia & Analgesia in terms of publications linked to the IOM report.

**Table 3:** The top 10 journals in which To Err is Human was cited.

**Source**	**Articles**	***h*-index**	***g*-index**	**TC**
Anesthesia and analgesia	60	22	37	1,494
Anesthesia	33	16	33	1,101
Anesthesiology	31	18	31	1,893
Current opinion in anaesthesiology	31	12	22	526
International anesthesiology clinics	28	4	8	88
Anesthesiology clinics	27	9	17	314
British journal of anesthesia	27	12	27	731
Journal of perianesthesia nursing	27	6	12	165
Anaesthesist	21	9	13	189
Aana journal	20	7	9	112
Canadian journal of anesthesia	17	8	15	245
Best practice and research: clinical anaesthesiology	15	10	15	413
Acta anaesthesiologica scandinavica	14	10	14	851
Annales francaises d'anesthesie et de reanimation	14	6	8	70
Pediatric anesthesia	14	5	9	98
Journal of clinical anesthesia	11	7	11	190
Anasthesiologie, intensivmedizin, notfallmedizin schmerztherapie	10	3	7	57
Advances in anesthesia	8	2	3	14
Indian journal of anesthesia	8	2	4	21
Quality and safety in health care	8	7	8	1,292

The productivity and citation impact of the sources was measured with the *h*-index and the *g*-index.

TC, total citations that articles received.

### Global distribution and cooperation

The origin of 867 documents (83.7%) could be linked to 49 different countries, while 16.3% of documents were devoid of geographical information. Institutions from 3 countries (USA, Germany, UK) were responsible for most of the scientific output (68%), while most countries contributed with only one to five articles (Table 4).

**Table 4:** Table, corresponding author's country, publication activity and frequency of international collaboration (2000–2019).

	**Country**	**Articles**	**Freq (%)**	**SCP**	**MCP**	**MCP-ratio**	**TC**
1	USA	475	54.79	451	24	0.0505	14,408
2	Germany	60	6.92	53	7	0.1167	728
3	United Kingdom	53	6.11	45	8	0.1509	2,627
4	Canada	39	4.5	32	7	0.1795	2,183
5	Switzerland	34	3.9	24	10	0.2941	942
6	New Zealand	27	3.1	19	8	0.2963	957
7	France	21	2.4	19	2	0.0952	530
8	Australia	20	2.3	15	5	0,25	306
9	Norway	14	1.6	12	2	0.1429	629
10	Israel	12	1.4	11	1	0.0833	144
11	Netherlands	12	1.4	12	0	0	265
12	India	11	1.3	11	0	0	27
13	Italy	9	1.04	8	1	0.1111	23
14	Spain	8	0.9	8	0	0	97
15	Sweden	5	0.6	5	0	0	69
16	China	4	0.5	3	1	0,25	30
17	South Africa	4	0.5	4	0	0	24
18	Thailand	4	0.5	4	0	0	86
19	Belgium	3	0.34	3	0	0	30
20	Brazil	3	0.34	2	1	0.3333	103

Freq (%) Percentage of all documents with information on country SCP, Single-Center Publications; MCP, Multi-Center Publications; MCP-Ratio, Number of MCP divided by number of articles. TC, Total citations.

**Figure 5:**
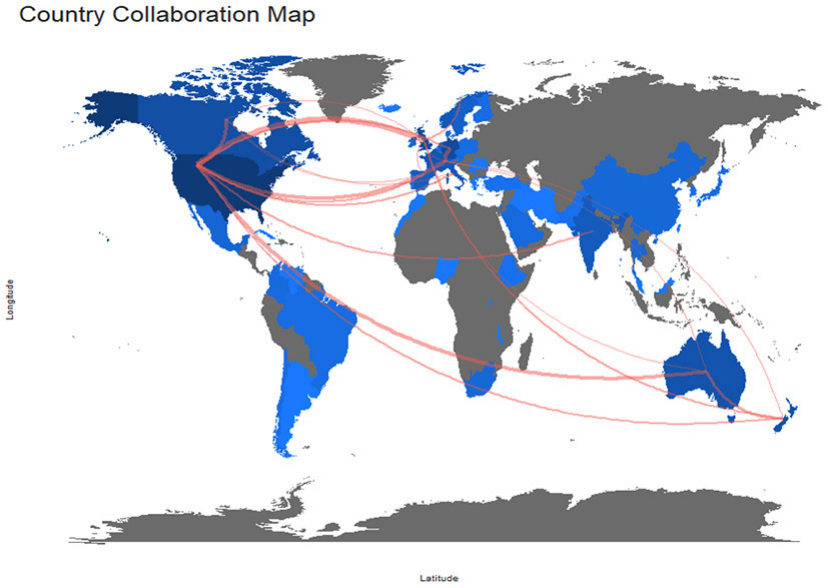
Map of international collaborations between 2000 and 2019. Every line represents a collaboration between institutions in two different countries. To better illustrate the pattern, only national collaborations with more than 10 joint publications in both decades are shown. The color intensity of blue is proportional to the number of publications of the country. Countries in gray did not publish any article that references *To Err Is Human*.

Most documents were published by a single academic center (SCP), while multi-center publications (MCP) made up only a small fraction of publications. The US features both: the highest publication activity and the lowest international collaboration rate (MCP-ratio of 0.05). As visualized in Figure 5, most collaboration existed between North America, Europe, and Australia. Less contribution could be identified for authors from Latin America, Africa, Asia, and India.

Regarding the institutional affiliations, the leading academic institutions were mostly located in North America, the most influential being Harvard Medical School (Table 5).

**Table 5:** The top 20 leading institutions regarding publications and citations liked to the IOM report.

	**Affiliations**	**Articles**
1	Harvard medical school	57
2	University of Toronto	53
3	University of Auckland	34
4	University of California	32
5	Johns Hopkins University	25
6	Johns Hopkins University School of Medicine	21
7	Massachusetts general hospital	18
8	University of Pennsylvania	17
9	Brigham and Women's Hospital	16
10	Virginia Commonwealth University	15
11	Vanderbilt University Medical Center	15
12	University of Washington	14
13	University of Chicago	14
14	Mayo clinic	14
15	University of Ottawa	13
16	University of Miami	12
17	Stanford University	12
18	Mcgill University	12
19	Haukeland University Hospital	12
20	Washington University School of Medicine	11

### Relevance and citation analysis

The analysis of citation and co-citation patterns of documents is of interest because a significant contribution to a scientific field can be assumed for frequently quoted papers. Also, papers that are more often cited together by other publications are more likely to relate to a shared subject area (8). Consequently, a change over time in the most locally cited references may be indicative of a shift in the thematic priority of patient safety. Table 6 lists the 20 most locally cited references between 2000 to 2019. To add granularity and better interpret thematic developments over time, Table 7 shows the 20 most local cited references in 5-year increments.

**Table 6:** The 20 most locally cited references within the anesthesiologic dataset between 2000 and 2019.

	**Cited document**	**Total local citations**	**Total global citations**
1	(18)	45	784
2	(19)	40	190
3	(20)	39	255
4	(21)	23	557
5	(22)	23	201
6	(23)	22	155
7	(18)	22	394
8	(24)	21	126
9	(25)	21	375
10	(26)	21	396
11	(27)	18	88
12	(28)	18	388
13	(29)	17	250
14	(30)	17	372
15	(31)	16	86
16	(32)	16	186
17	(33)	16	90
18	(34)	14	734
19	(35)	13	140
20	(36)	12	33

The IOM report “*To Err is Human”* was not included in this dataset and therefore didn't need to be excluded.

**Table 7:** The 20 most locally cited references within the anesthesiologic dataset between 2000 – 2019, sorted in 5-year increments for higher granularity.

	**2000–2004**		**2005–2009**		**2010–2014**		**2015–2019**	
	**Cited reference**	**TC**	**Cited reference**	**TC**	**Cited reference**	**TC**	**Cited reference**	**TC**
1	(2)	11	(2)	11	(37)	19	(37)	14
2	(38)	10	(38)	10	(38)	11	(2)	7
3	(4)	9	(39)	10	(25)	11	(40)	7
4	(41)	9	(42)	10	(43)	9	(44)	7
5	(45)	7	(46)	8	(21)	9	(45)	7
6	(47)	6	(4)	7	(32)	8	(48)	6
7	(43)	6	(45)	7	(49)	8	(2)	6
8	(50)	6	(4)	6	(51)	8	(20)	6
9	(52)	5	(43)	6	(2)	7	(26)	6
10	(53)	5	(18)	6	(37)	7	(49)	6
11	(54)	5	(19)	6	(55)	7	(24)	5
12	(2)	5	(25)	5	(56)	7	(57)	5
13	(58)	5	(54)	5	(18)	7	(59)	5
14	(60)	5	(61)	5	(22)	7	(28)	5
15	(62)	5	(63)	5	(64)	7	(65)	5
16	(66)	4	(56)	5	(39)	7	(67)	5
17	(68)	4	(20)	5	(69)	7	(70)	5
18	(71)	4	(72)	5	(51)	7	(73)	4
19	(74)	4	(29)	4	(75)	6	(76)	4
20	(77)	4	(78)	4	(47)	6	(79)	4

TC, Total Citations.

While published long before the IOM-report, “An analysis of major errors and equipment failures in anesthesia management: considerations for prevention and detection” by Cooper et al. (38) remained the most (2000–2009) or second-most (2010–2019) cited article in the anesthesiologic dataset. A discursive shift toward increasing interest in checklists is manifested by the Safe-Surgery-Saves-Lives-Study-Group paper “A surgical safety checklist to reduce morbidity and mortality in a global population” (37), showing the most local citations in the 2010–2019 period. When examining the 20 most relevant authors, David Gaba is by far the most influential contributor with 613 local citations (Table 8). The perceived predominance of certain theoretical frameworks in anesthesia, such as the “Swiss Cheese” metaphor for system failure and “Non-technical skills/CRM” are underscored by the high ranking of authors like James Reason and Rhona Flin.

**Table 8:** Overview of the 20 most locally cited authors within the anesthesiologic dataset.

	**Year**	**2000–2004**	**2005–2009**	**2010–2014**	**2015–2019**	**2000–2019**	**% of** **references**
	Total references	4.741	9.729	14.347	11.211	38.515	100
	**Researcher**	**Citations revieved**					
1	Gaba DM	144	174	158	137	613	1,6
2	Leape LL	97	127	129	77	430	1,1
3	Cooper JB	78	89	102	85	354	0,9
4	Bates DW	57	121	90	55	323	0,8
5	Merry AF	46	69	116	83	314	0,8
6	Brennan TA	61	69	108	53	291	0,75
7	Howard SK	56	80	70	72	278	0,72
8	Flin R	20	49	109	87	265	0,69
9	Webster CS	39	61	83	71	254	0,66
10	Runciman WB	61	72	70	37	240	0,62
11	Helmreich RL	26	87	77	47	237	0,61
12	Reason J	51	82	53	43	229	0,59
13	Vincent C	14	52	93	61	220	0,57
14	Pronovost PJ	6	27	119	57	207	0,54
15	Thomas EJ	26	65	71	44	206	0,53

The authors of the IOM report *To Err is Human* were excluded.

### Co-word analysis and thematic evolution

One of the aims of this study is to identify and visualize the main concepts that patient safety researchers in anesthesiology connected with the IOM report and to analyze how research content and orientation might have expanded or narrowed over time. To best capture the scientific content stored in the database, the parameters ”author keywords,” “Keywords Plus,” “titles of publications,” and “abstracts” were applied to compare the top twenty words of the sub-periods. We aggregated interchangeable terms into a single primary category (e.g., “errors,” “error,” “medical error”).

A comparison of *Keywords Plus* showed a 64.5% overlap of key terms. The most common terms were: “adult,” 84anesthesia,” “anesthesiologist,” “male,” “female,” “human(s)”, “healthcare quality,” “United States,” and “patient care.” However, beginning in 2010 two new terms emerged: “education” and “simulation training.” The term “clinical competence” was present since 2004, which is in stark contrast to non-anesthesiologic patient safety literature (7).

A comparison of the *abstract words* showed an 62.5% overlap of key terms within all four data bases. The most common terms were “an(a)esthesia,” “adverse,” “clinical,” “care,” “error(s)“, 84events,” “patient(s)”, “training,” and “health”.

The top twenty *title words* showed a 55% overlap, with the most common terms being “safety”, “care,” “patient,” “management,” “errors,” “simulation” and “training.” From 2010 onward, the terms “perioperative,” “review,” “checklist,” “team” and “practice” signify a thematic development. This also applies to *Author keywords* (63% overlap), which displayed a thematic evolution: “patient safety,” “education,” “crisis management” and “simulation” were supplemented by “perioperative care,” “quality improvement” and “teamwork” starting in 2010.

**Figure 6:**
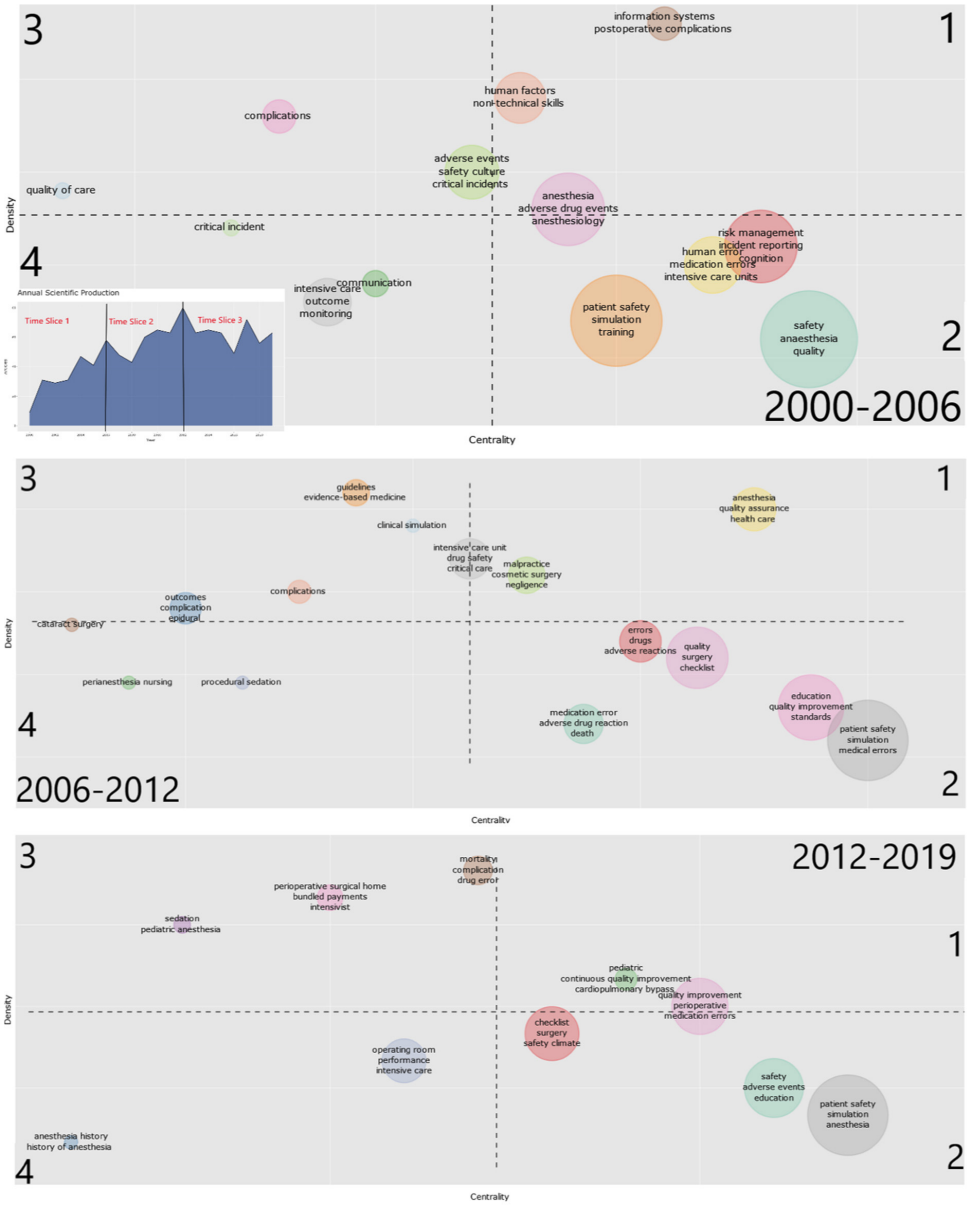
Strategic diagram of the 2000–2006, 2006–2012, and 2012–2019 sub-periods. The numbers indicate the type of clusters defined by their centrality and density as proposed by Michel Callon (80). Each cluster is labeled with the corresponding three most frequent keywords within the cluster. *Inserted figure:* Distribution of documents per year with 2 cutting points at the two publication peaks in 2006 and 2012. The cluster colors were randomly assigned by biblioshiny and therefore differ across subperiods.

Based on the keywords that authors provide to characterize their research, it is possible to create a co-occurrence network that reveals the conceptual structure of a research area and to visualize them on a strategic diagram with its two dimensions of centrality and density (80). While *centrality* measures the intensity of a given cluster's links with other clusters, *density* characterizes the strength of the links that tie the words making up the cluster together. All clusters can be divided into four general categories by ordering them horizontally (along the x-axis) by increasing order of centrality, and vertically (along the y-axis) by increasing order of density (Figure 6). If the time span is futher differentiated into different time slices, a dynamic analysis based on the synthetic and simplified presentation of the network's morphology is possible.

During the first period (2000–2006, Figure 6), the most highly developed cluster that had been dealt with systematically over a longer period (quadrant 1) is research on information systems and postoperative complications as well as adverse drug events. Central themes, but with weaker internal correlations, are quality and safety issues in anesthesia. The study of error in a variety of forms and settings and the role of incident reporting are emerging as central themes with weaker internal correlations (quadrant 2), underscoring the pioneering influence of anesthesia when compared to a more general medical database (7).

During the second sub-period (2006–2012, Figure 6), previously underdeveloped topics started to become more central to the scientific debate, such as medication safety and intensive care; the latter being catapulted from decentralized and poorly recognized subject to focus of contested debate within only a few years. Moreover, publications concerning the use and merit of guidelines and evidence-based medicine emerged as strongly linked and became more central.

In the third sub-period (2012–2019, Figure 6), yet another discursive shift can be recognized, with quality-related topics (e.g., management, improvement, performance) more central than ever to the scientific debate. The cluster comprising simulation and medical education remains important, however many of the previously found topics closely associated (e.g., non-technical skills, human factors, safety culture) are no longer represented. Almost two decades after the release of *To Err Is Human*, “errors” remain central to the debate (quadrant 1 and 2), with an emphasis on medication errors. Also, perioperative communication, often linked to checklists, retain an important position in the scientific landscape, although many publications exist since 2004. Interest in other core topics previously in the spotlight, like intensive care, fades quickly and is seemingly replaced by a more managerial agenda.

## Discussion

The current study aimed to gain an understanding of the impact of the IOM report *To Err Is Human* on patient safety research in anesthesiology, to identify the research activity explicitly related to this seminal publication and to see how the ideas presented in *To Err Is Human* might have affected the diversity of the safety science discourse in anesthesiology over the last 20 years. Therefore, bibliometric methods were applied to a data set which was linked to the IOM report through a reference (i.e., bibliographic coupling). This constitutes an established methodological approach in bibliometric literature, where references are understood to represent an author's conscious decision to express a relationship between his own manuscript and the cited document (81), rather than a random event or mere bibliographic data at the end of a manuscript.

Core themes and resulting changes in the patient safety discourse over time were visualized on a strategic diagram by applying a clustering algorithm on the co-occurrence network of author keywords. One noticeable result of our analysis is the observation that “errors,” while initially a core topic in the scientific discourse within the anesthetic database, are subsequently replaced by terms representing a somewhat more granular, team-oriented and educational approach. Contrary to the perceived tendency of healthcare to reframe the problem of medical harm into the problem of “human error” as an objectively identifiable, measurable and countable, unique category of human performance (7), these findings underscore a central argument of Wears and Sutcliffe (1) about “the special case of anesthesia” within the patient safety discourse: In the two decades preceding the IOM report, anesthesiologists had already developed substantive and sustained partnerships with the safety sciences. Human factors professionals were not merely consultants on a clinical research project but rather embedded within departments, combining forces with clinicians to learn about managing the complexities and risks of anesthetic practice (82, 83). In following this research tradition and contrary to the broader patient safety movement, anesthesia seemed less distracted by “fruitless and sterile” efforts of eliminating errors (1), likely influenced by the emerging consensus among safety scientists that errors had to be interpreted rather as symptoms than causes and were representative of deeper trouble within complex adaptive systems that required further investigation rather than elimination (3, 84). When comparing the data from the co-word analysis with the arguments of *To Err is Human*, this is remarkable, as the initial development within anesthesia was in line with the IOM report's stated objective of moving the focus from individual errors to systemic issues; adverse events were understood as a property of a system of care rather than the result of deficient health care professionals.

However, our findings also indicate that patient safety related research in anesthesia, while profiting from a certain intellectual and conceptual head start, was not immune to a discursive shift toward more managerial, quality-management related topics as observed in the health care system as a whole (7). These topics were representative for a type of scientific-bureaucratic medicine with strong conceptual roots in public health and epidemiology and the explicit valuing of aggregate data over individual cases, as exemplified by the movements for clinical practice guidelines and evidence-based medicine.

Consequently, the mainstream patient safety movement seems to have gradually taken over safety approaches in anesthesia, dominated by a narrative of competence and control that implicitly pushed back on outside intervention (85), effectively silencing the diversity that had created progress in the first place.

The most frequently cited safety scholar in our database was James Reason (39, 45). While the IOM report mentions a variety of safety theories and frameworks, the heavy emphasis on Reason's work together with a tendency of medical professionals to oversimplify theoretical foundations in safety science might have inadvertently contributed to what can be characterized as a consolidation of the error narrative. The models proposed by Reason (39, 45, 86, 87) provided medical metaphors (e.g., resident pathogens) as well as memorable graphical representations (e.g., Swiss Cheese Model) that resonated well with health care providers, and created the impression of an intuitive simplicity which made clinicians believe that they had understood the model when in fact they hadn't (88). Instead of developing a systems approach in healthcare based on the systemic aspects of Reason's framework (89), patient safety research by clinicians ended up becoming just another attack on “human error,” a focus implicitly encouraged by the title of James Reason's bestselling book.

Also, it is surprising to note the low presentation of research on incident reporting. Given the importance the IOM report placed on mandatory and voluntary reporting systems, it is remarkable that only the early years after publication of the IOM report show much research focusing on incident reporting or reporting systems. We explicitly checked the frequency of the term “reporting” in the document titles during all four sub-periods to compensate for limitations with relying on author keywords, however the results confirmed the initial findings that research on incident reporting was not a key concern in any of the clusters. As with the discussion about research traditions, it again seems as if anesthesia, after a perceived head start due to independent research efforts during the 1980's and 1990's, had become increasingly aligned with a broader patient safety agenda dominated by health professionals focused on programmatic activity that was more concerned with the large-scale transfer of interventions successful in other fields (e.g., checklists from aviation) than the contextual understanding of their internal mechanisms (90).

### Limitations

The main limitation of this study, both regarding the records identified through database searching and in thematic diversity, is that the data set was limited to documents referencing *To Err Is Human*. Therefore, the results do not claim to portray a comprehensive picture of all the research on patient safety done since *To Err Is Human* was first published, but rather reflect the analysis of a specific subgroup of those authors that have identified and referenced a connection between their work and the arguments presented in the IOM report. This is based on the assumption that a reference to the IOM report in our data base represents the conscious decision made by an author to connect a particular argument in the document he was writing to the work he was citing (81). Despite all the arguments in the bibliometric literature claiming reliable reference motives, it is conceivable that many authors cited the publication of *To Err is Human* as global representation of a new era in patient safety research rather than referring to a specific argument within the IOM report. In this case, the point of reference would not be the content of the report, but rather the historical impact it had on subsequent years. Unfortunately, the bibliometric method itself does not allow any discrimination between both possibilities.

Another potential limitation regarding the analysis of the academic discourse on patient safety is the inclusion of other documents such as book chapters, conference papers, and editorials rather than peer-reviewed manuscripts only in our data base. This approach was chosen to gain a comprehensive impression of the academic impact as reflected in the variety of written communications that constitute the exchange and development of scientific ideas. A focus on the assessment of the academic quality would have made the restriction to high-quality, peer reviewed journals mandatory.

## Conclusion

The current study contributes to an understanding of the seminal IOM report's scientific impact on patient safety research in anesthesiology from 2000 to 2019. During this period, the research context expanded from the initial focus set forth by the report, which ultimately led to an underrepresentation of research on a systems approach and incident reporting. Important collaborations with safety researchers from outside of health care dating back to the 1990's were gradually reduced, while previous research within anesthesiology was aligned with a broader, more managerial patient safety agenda. For future safety efforts, anesthesiologists might be well-advised to rekindle these old collaborations to resume a vanguard role in patient safety research. This will also entail renewed discussions about the understanding and role of “human error,” harnessing expertise from domains outside of healthcare, and a reluctance to simplify human interactions within complex systems into easily digestible bites that might suit safety campaigns and managerial agendas but fall short in addressing the needs of patients and practitioners alike.

## Data availability statement

Publicly available datasets were analyzed in this study. This data can be found here: www.scopus.com; using the search terms as specified in the methods section.

## Author contributions

CN: conceptualization, formal analysis, validation, and writing—original draft. PG: investigation, data curation, and formal analysis. JB: conceptualization, supervision, and writing—review and editing. MS: conceptualization, validation, supervision, and writing—review and editing. All authors contributed to the article and approved the submitted version.

## Funding

For the publication fee we acknowledge financial support by Deutsche Forschungsgemeinschaft (DFG) within the funding programme Open Access Publikationskosten as well as by Heidelberg University.

## Conflict of interest

The authors declare that the research was conducted in the absence of any commercial or financial relationships that could be construed as a potential conflict of interest.

## Publisher's note

All claims expressed in this article are solely those of the authors and do not necessarily represent those of their affiliated organizations, or those of the publisher, the editors and the reviewers. Any product that may be evaluated in this article, or claim that may be made by its manufacturer, is not guaranteed or endorsed by the publisher.
